# A Glance at Biogenesis and Functionality of MicroRNAs and Their Role in the Neuropathogenesis of Parkinson's Disease

**DOI:** 10.1155/2023/7759053

**Published:** 2023-06-08

**Authors:** Adam Szelągowski, Mariusz Kozakiewicz

**Affiliations:** Nicolaus Copernicus University in Toruń Ludwik Rydygier Collegium Medicum in Bydgoszcz, Faculty of Health Sciences, Department of Geriatrics, Bydgoszcz, Poland

## Abstract

MicroRNAs (miRNAs) are short, noncoding RNA transcripts. Mammalian miRNA coding sequences are located in introns and exons of genes encoding various proteins. As the central nervous system is the largest source of miRNA transcripts in living organisms, miRNA molecules are an integral part of the regulation of epigenetic activity in physiological and pathological processes. Their activity depends on many proteins that act as processors, transporters, and chaperones. Many variants of Parkinson's disease have been directly linked to specific gene mutations which in pathological conditions are cumulated resulting in the progression of neurogenerative changes. These mutations can often coexist with specific miRNA dysregulation. Dysregulation of different extracellular miRNAs has been confirmed in many studies on the PD patients. It seems reasonable to conduct further research on the role of miRNAs in the pathogenesis of Parkinson's disease and their potential use in future therapies and diagnosis of the disease. This review presents the current state of knowledge about the biogenesis and functionality of miRNAs in the human genome and their role in the neuropathogenesis of Parkinson's disease (PD)—one of the most common neurodegenerative disorders. The article also describes the process of miRNA formation which can occur in two ways—the canonical and noncanonical one. However, the main focus was on miRNA's use in in vitro and in vivo studies in the context of pathophysiology, diagnosis, and treatment of PD. Some issues, especially those regarding the usefulness of miRNAs in PD's diagnostics and especially its treatment, require further research. More standardization efforts and clinical trials on miRNAs are needed.

## 1. Introduction

The second half of the twentieth century brought significant discoveries in the field of molecular biology, among which one of the most significant one was the discovery of many types of small RNA molecules (sRNA), later classified as noncoding RNA (ncRNA), such as tRNA, rRNA, uRNA, snoRNA, snRNA, or siRNA. This group also includes numerous and commonly found in living organisms microRNAs (miRNAs). The common feature of ncRNAs is that, unlike mRNAs, they do not encode specific proteins. Currently, it is believed that their key role in the genome is a highly specific recognition of nucleic acid sequences, then directing the regulation of gene expression (transcription, RNA interference, and epigenetic processes), and mediating the introduction of RNA modifications. They are also assigned the role of specific “molecular fuses,” responsible for recognizing and directing the destruction of foreign RNA formed inside the cell during viral infections or in the course of cancerogenesis. Abnormalities in the level of expression of specific miRNAs, but also defects in proteins that build complexes, participating in the biogenesis of these molecules and ensuring their full functionality in the genome, are increasingly found in the course of many human diseases. miRNAs are characterized as both more commonly found and more stable in biological samples than other ncRNAs. Changes in miRNAs' concentrations in pathological conditions usually seem to be highly tissue specific. This facilitates their use in in vitro and in vivo studies in the context of diseases' pathophysiology, diagnosis, and treatment. The central nervous system (CNS) is the most abundant source of miRNA transcripts in living organisms [1–3]. In this review, we present a current knowledge on a biogenesis and functionality of the miRNAs in human genome and their role in the neuropathogenesis of the second most common neurodegenerative disorder, namely, Parkinson's disease. Our objective was also to perform the latest literature review, regarding the potential use of miRNAs in PD's diagnostics and therapy.

### 1.1. Characteristics and Organ Specificity of miRNAs

Lin-4 was the first the microRNA discovered in 1993 by Ambros et al. Cel-lin-4 miRNA was detected during research conducted on the heterochronous Lin-14 gene in the *Caenorhabditis elegans* nematodes. It was complementary to the sequence in the 3′ UTR region of lin-14 mRNA, and it had an inhibitory effect on the accumulation of the lin-14 protein in worm cells. This regulation is indispensable in synchronizing the larval development of *C. elegans* [4]. In the following years, a structurally nonhomologous, but cell-lin-4-like miRNA molecule cel-lin-7, was also identified in the same organism, which was then also detected in vertebrates, including humans, but also tunicates, molluscs, annelids, arthropods, and half-strings. This has indicated the existence of a specific system of gene expression regulation within the genomes of numerous species [5, 6].

The length of miRNA molecules varies, according to different authors, in the range of 17-25 nucleotides. Currently, it is believed that up to 40% of the miRNA-coding sequences in mammals are located within introns (so-called miRtrons) and exons of genes encoding various proteins of the body, and about 10% within the introns of long transcripts for ncRNA. Nucleotide sequences for individual miRNAs can overlap within the genome, and their original transcripts may undergo an alternative splicing process in the host body, but the origin of many of these molecules remains unknown [7]. So far, nearly 2,000 miRNA molecules have been characterized in *Homo sapiens*. Their registry, created and developed by the team of Prof. Sam Griffiths-Jones, now functions as a miRbase database at the online address of http://www.mirbase.org [8, 9]. Total miRNA count in the human genome is estimated at several thousand. According to various literature reports, those transcripts can affect the expression of 30-60% of the genes encoding the body's proteins. One specific type of microRNA can regulate many genes, and one gene can be regulated by numerous molecules [2, 3].

The discovery of further miRNAs made it possible to assign them to tissue-specific panels. It was quickly noticed that the expression of specific miRNA molecules undergoes dynamic changes not only in pathologically altered tissues, in inflammation, cancerogenesis, and neurodegenerations but also in physiological processes, for example, in the course of cell apoptosis [10–12]. In 2008, Mitchell et al., using the method of radioactive labeling with the isotope ^32^P and PAGE, detected the presence of short RNA molecules in human blood plasma, among which numerous sequences with a length of approximately 22 nucleotides were identified. They were not susceptible to DNase I digestion and were sensitive to RNase, thus corresponding to the characteristics of miRNAs. These were later called circulating extracellular miRNAs (ECmiRNAs). In later stages, the authors proved that plasma concentrations of molecules such as miR-15b, miR-16, and miR-24 closely correlate with those observed in blood serum. Then, using a model of a human prostate cancer cell xenograft 22Rv1 on 12 mice with induced NOD/SCID immunodeficiency, Mitchell et al. showed that serum concentrations of miR-629 and miR-660 allowed to distinguish xenograft mice from healthy mice with almost 100% sensitivity and specificity. They also moderately correlated with a tumor mass [13]. In later years, the presence of a wide range of ECmiRNAs was also confirmed in many body fluids, such as urine, saliva, peritoneal fluid, amniotic fluid, bronchial washings, or cerebrospinal fluid. In the course of further research, organ-specific differences in miRNA concentrations in different diseases were observed. The existence of circulating, exogenous miRNAs, produced and secreted by the intestinal microflora, the profile of which may vary interindividually depending on the diet, has also been confirmed; however, their role still remains unclear. It seems that while intracellular miRNA molecules play an indispensable role in the functioning of the genome of many living organisms, ubiquitous, circulating miRNAs can be direct or indirect activators of many intercellular signaling pathways. An important property of circulating miRNAs is their high stability compared to intracellular miRNAs—thanks to the inclusion of miRNAs in lipid vesicles and the ability of binding to proteins, they are highly resistant to denaturation or RNases. Circulating miRNAs are characterized by a high durability in biological samples. They maintain a long-term stability at a room temperature and are resistant to a multiple freezing and thawing cycles [3, 12–15].

All the previously mentioned features of circulating miRNA molecules cause that in recent years, attempts to create organ-specific diagnostic panels, where various miRNAs could act as biomarkers, have been made. Changes in their expression and changes in the activity of enzymes involved in the biogenesis of miRNA have been characterized in the course of many human diseases. Fluctuations in specific miRNA concentrations in malignant neoplasms of many organs have been characterized. miRNAs there can act as both inducers and suppressors of carcinogenesis. Their expression can be increased or decreased depending on the type of miRNA molecule [16–18]. Abnormal miRNA expression profiles are also found in the course of cardiovascular diseases, diabetes, sepsis, or nervous system diseases, including neurodegenerations. This is particularly important since up to 70% of the body's miRNA pool occurs within the CNS, where these molecules play a key role in its pre and postnatal development. It is assumed that in the pathogenesis of neurodegeneration, especially in the context of the accumulation of pathogenic peptides and proteins resulting in neuronal dysfunction, miRNA molecules may play a significant role [19–25]. Nowadays, Alzheimer's disease and Parkinson's disease in particular are becoming an increasingly common and growing clinical problem, with the increase in life expectancy and the aging of the populations of highly developed countries. They are also a major diagnostic challenge, especially due to the relatively low specificity and high invasiveness of many of the procedures currently used [26, 27].

## 2. Biogenesis and the Mechanism of Action of miRNA Molecules

miRNAs are formed in two pathways, called canonical and noncanonical, depending on the involvement of individual processor proteins at various stages of the formation of a given molecule, the location of the sequence of a given miRNA in the genome, or the participation of other mechanisms in the formation of a functional molecule. Some miRNAs may be the end product of both pathways. Still, many mechanisms related to the functioning of various variants of the noncanonical pathway, as well as the interaction of miRNAs with the host genome, remain unexplained.

### 2.1. The Canonical Pathway and the Formation of the miRNA-Induced Silencing Complex (miRISC)

**Figure 1:**
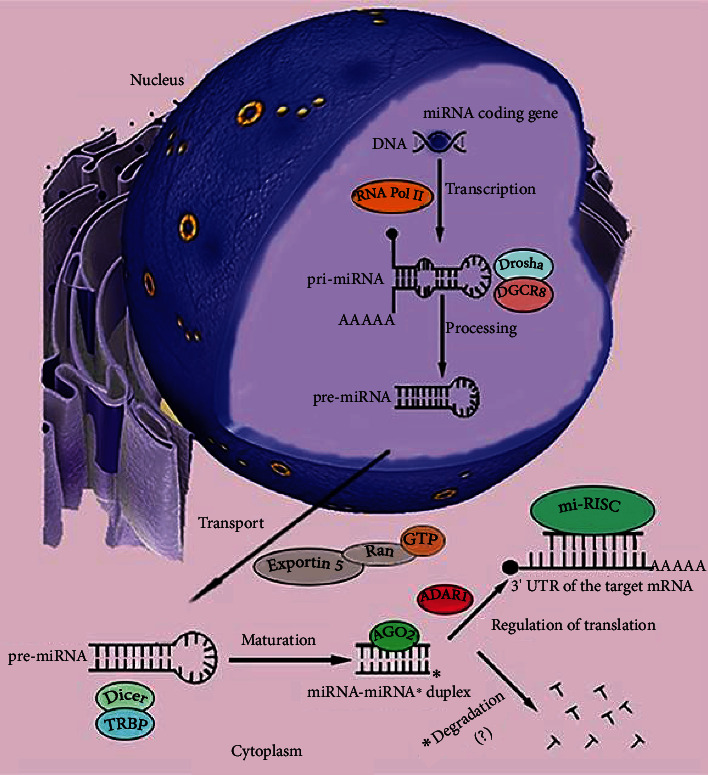
Canonical pathway of the microRNA (miRNA) formation. Genes for miRNAs are transcribed in the cell nucleus mainly by the RNA polymerase complex (RNA Pol II). As a result, long primary miRNA transcripts (pri-miRNA) of hairpin structures are formed. The 5′ pri-miRNA end has a guanylate cap, but the 3′ end is not always polyadenylated. The microprocessing complex of DGCR8 and Drosha proteins directs the treatment of pri-miRNA, which leads to the release of the 5′ and 3′ end of the transcript and its conformational changes. The resulting precursor miRNAs (pre-miRNAs) are recognized via the 3′ free end and transported through the nuclear pores to the cytoplasm of the cell by the Exportin-5 and Ran-GTP complex. In the cytoplasm, pre-miRNA is bound by a complex of Dicer and TRBP proteins. Due to the activity of RNase III Dicer, the transcript loses its loop structure. Duplexes of complementary strands are formed, the leading and the passenger (miRNA-miRNA^∗^) with a length of about 22 nucleotides. They are targeted by the elements of the RISC complex, i.e., Argonaut proteins such as AGO2. Thanks to the activity of Slicer endonuclease, AGO2 is able to separate the duplex strands. The proportions of binding as the leading strand a 5′ or 3′ miRNA strand to the AGO protein in RISC is variable. They appear to be tissue-specific and depend on their nucleotide structure. The leading strand is loaded on RISC. This process is mediated by the ADAR1 protein. The resulting full-functional processor complex (miRISC), when bound to the 3′ UTR region of the target mRNAs, becomes a powerful regulator of gene expression. The miRNA^∗^ strand, deprived of the protective effect of the complex proteins, usually undergoes enzymatic degradation. Some miRNAs^∗^ are detected in the cells and body fluids of organisms. Their role remains unclear.

In the canonical way, long primary miRNA transcripts (pri-miRNA) are formed through transcription usually with the participation of RNA polymerase II, forming the structure of a hairpin and having a 5′ guanylate cap at their end. Not all of the transcripts contain polyadenylated 3′ end. Pri-miRNA is then recognized and processed by a microprocessor complex of the proteins Drosha (showing RNase III activity) and DGCR8 (Di-George's syndrome critical region protein, also known as Pasha). This results in the formation of precursor mi-RNAs (pre-miRNAs), having a length of 60-70 nucleotides. These molecules have, in their structure a characteristic, unpaired dinucleotide 3′ overhang. This motif allows pre-miRNA to be attached by a highly specific protein complex: Eksportine-5 (XPO5)-RanGTP, providing it with protection against degradation and transporting it through the nuclear pores to the cytoplasm. Outside the nucleus, the miRNA molecule is recognized and further processed by a complex of DICER proteins and TRBP (transactivation response element RNA-binding protein) or PACT protein-homologues. DICER, having RNase III activity, cuts off the loop from the pre-miRNA structure, which leads to the formation of miRNA/miRNA^∗^ duplexes, which are about 22 nucleotides long, with the 3′ end motif preserved. One of the strands is called the leading (guide), and the second one—the passenger. Then, there is an interaction of miRNA/miRNA^∗^ molecules with the RISC complex, which includes such proteins as DICER, TRBP, or Argonaut 2 protein (AGO2), which consists of the PIWI domain and shows the activity of Slicer endonuclease. This leads to cutting, destabilization, and separation of miRNA/miRNA^∗^. Other enzymes, i.e., helicases, can also be involved in this process. The quantitative ratio, in which the AGO proteins of the RISC complex are present in the cytoplasm of the cell in combination with the 5′ or 3′ strand of the duplex of a given miRNA molecule, may be different. It shows tissue specificity and significant variability, especially in many pathological conditions [28–33]. The affinity of AGO proteins for individual strands may vary. It is significantly influenced by a primary structure of duplex transcripts, which affects their thermodynamic stability. In the human genome, duplexes containing uracil nucleotide at the 5′ end, with a predominance of purines (A/G) in the structure function as guide strands. In passenger strands, at the 5′ end, cytosine is the most common nucleotide, and pyrimidine bases (U/C) predominate in a whole sequence [33, 34]. The leading strand is incorporated into the RISC complex, forming miRISC, and it is mainly in this form that it plays the role of a regulator of genetic expression. Loading of the miRNA molecule into the RISC complex and its activity is supported by the adenosine deaminase RNA-specific (ADAR1) protein. The knockout of the ADAR1 gene in mouse embryos results in global inhibition of miRNA expression and the formation of a potentially lethal phenotype [35]. The separated passenger strand is most often a target for proteins such as TRAX, TRANSLIN, or HSP90 chaperones, forming the C3PO complex. Endonuclease activity of the C3PO causes miRNA^∗^ strand degradation. Some types of miRNAs^∗^ are detected both physiologically and in the course of various diseases, but the role they play and how they interact with the leading strands in miRISC remain unclear (Figure 1) [27–32].

Newly created miRISC complex attaches to the target sequence in the 3′ UTR region of mRNA in the base pairing mechanism. In this process, AGO proteins again play a significant role, in which the spatial structure of functional domains such as PIWI dynamically changes, depending on the stage of RNA interference, as evidenced by crystallographic studies [36, 37]. The main role of miRISC is to inhibit the expression of the target gene, although there are reports of reverse situations, in which positive gene expression can be regulated through miRISC by protecting the miRISC-associated mRNA from degradation [38].

Currently, there are two ways in which miRISC interacts with target mRNAs. Hydrolytic mRNA cutting by miRISC can only be done if the AGO protein of the complex has a PIWI domain. Only the AGO2 protein has this property, out of the at least eight AGO proteins characterized so far in the human genome. In addition, this process requires almost 100% complementarity of nitrogenous bases between the guide strand in the microprocessor complex and the target mRNA. A series of physicochemical interactions of the triad of two residues of aspartic acid and aspartate or histidine in the PIWI domain with magnesium cations and the water molecule cause the phosphodiester bond between mRNA nucleotides paired with specific strand nucleotides in miRISC to break. The result is a collapse of the mRNA reading frame, inhibiting its translation and making it susceptible to the effects of exonucleases. According to modern reports, animal miRNAs rarely show a degree of complementarity, allowing the use of a mechanism dependent on the activity of the slicer endonuclease of the PIWI domain [39–42].

**Figure 2:**
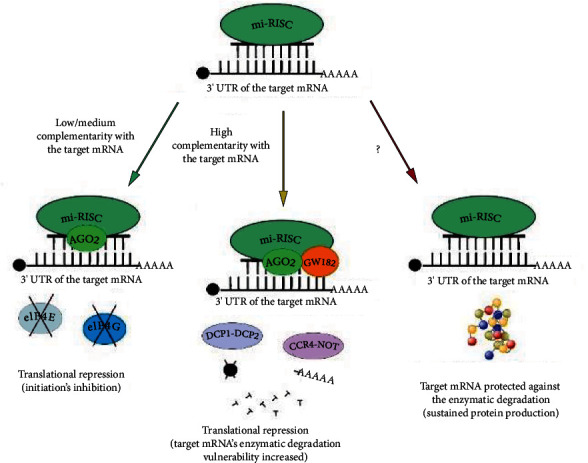
Regulation of gene expression by miRNA. The miRNA molecule in the miRISC complex may show varying degrees of complementarity with the target mRNA (green arrow). Low or medium pairing of nitrogenous bases (of the order of approx. 30-60%) leads to the activation of the translational repression mechanism by creating a spatial disturbance on the mRNA strand by the mi-RISC complex, preventing the interaction between the elF4E and elF4G translation initiation factors. This results in preventing the recruitment of ribosomal subunits and, as a consequence, inhibiting the biosynthesis of the protein, that is, the product of the target gene. The high complementarity of miRNA in miRISC and target mRNA (>80-90%, yellow arrow) and the presence of the AGO2 protein, as well as additional proteins and their complexes, such as GW182, DCP1-DCP2, and CCR4-NOT, enables the repression of translation by degradation of the mRNA transcript. After hydrolysis of its guanylate cap and deadenylation of the 3′ end, mRNA becomes susceptible to intracellular nucleases and enzymatic cutting by the proteins of the mi-RISC complex, mainly AGO2. The cut mRNA transcript becomes nonfunctional. In some cases, miRISC can attach target mRNAs, protecting them from nucleases and degradation (red arrow). This results in maintaining protein biosynthesis and increasing its expression in the cell.

A much more common mechanism of epigenetic modulation of genome expression by miRISC is translation repression. It requires a much lower degree of complementarity with the target mRNA (of the order of several base pairs) than in the case of enzymatic cutting, and its nature largely depends on the type of Argonaut protein, which cocreates miRISC. The process of translational repression, which has many similarities in mammals but has so far been best characterized in Drosophila, can proceed in essentially two ways. The first possibility includes the interference of the AGO2 protein of the miRISC complex in the interaction between the elF4E and elF4G translation initiation factors, which makes it impossible to create a functional initiation complex and recruit ribosomal subunits to the target mRNA. In the second case, when the elements of the microprocessor complex are the proteins AGO1 *Drosophila* and GW182, the repression of translation occurs as a result of the degradation of the transcript. The GW182 protein, by engaging both the CCR4-NOT and DCP1-DCP2 complexes into the target mRNA, induces, respectively, deadenylation and removal of the poly(A) tail from the 3′ end of the transcript and hydrolysis of the guanylate cap at the 5′ end. Three possible ways of the miRNA-mediated gene expression modulation are presented in Figure 2. Another function of the GW182 protein is to mediate the formation of P-bodies, and ribonucleic granules in which subjected to translational repression are detected but protected from the degradation of mRNA molecules. The role of P-bodies has not yet been fully explained [39, 43, 44].

### 2.2. Last, but Not Least: The Noncanonical Pathway of miRNA Formation

Since it has been described, the role of noncanonical miRNA molecules has been the subject of extensive discussion about their function in regulating genome activity and its consequences for the phenotype of the cell. This is largely due to the fact that transcripts of many genes have sequences complementary to the primary structures of miRNAs formed in a noncanonical mechanism. Literature reports distinguish their involvement in the induction of apoptotic processes in neurons, the development of insulin resistance, carcinogenesis, immune response, or stem cell differentiation. It is worth noticing, especially due to the fact that noncanonical miRNAs often show a quite little complementarity (e.g., 40% for miR-155) with the target region in mRNA, so their impact on gene expression may theoretically be limited compared to canonical molecules [45, 46].

Our knowledge of the contribution of noncanonical miRNAs to both the physiology and pathology of the human body remains limited. Several of the most frequently described and best-characterized mechanisms of formation of these molecules are presented below.

The first of the characterized noncanonical miRNA pathways is the mirtron pathway. The introns of the host genome, encoding sequences for miRNA molecules, were characterized in the first decade of the twenty-first century in *Drosophila* [47]. Pri-miRNAs derived from miRtrons, as in the canonical way, undergo splicing and form hairpin structures. However, they are characterized by a smaller length. This difference has consequences; these molecules cannot be identified and treated by the Drosha/DGCR8 complex. It is replaced at this stage by DBR1, the hydrolase that catalyzes the cutting reaction of the 2′, 5′ phosphodiester bonds of the mirtron's lasso structure, which releases spatially linear pri-miRNAs. Further stages of the biogenesis of miRNA molecules converge. The produced pre-miRNAs are moved from the cell nucleus to the cytoplasm by the XPO5-RanGTP complex and processed by the endoribonuclease complex DICER. Mature miRNA molecules are incorporated into RISC, similarly to the canonical way. The development of advanced bioinformatics tools in recent years has made it possible to distinguish mirtron miRNAs from canonical miRNAs, basing on subtle differences in their length and content of individual nitrogenous bases [48–51].

miR-451 is the only characterized molecule whose formation is not dependent on the activity of the full AGO/DICER/TRBP complex. It obtains its functionality after further processing by the AGO2 protein alone. The factors inducing this mechanism are the length of the transcript (miR-451 consists of only 17 nucleotides) and the occurrence of incorrectly paired bases in the structure of the miR-451 hairpin. In order for this variation of the noncanonical pathway to lead to the formation of a mature miRNA molecule, the presence of a human elF1A translation initiation factor in the AGO2-elF1A complex is also necessary. elF1A also remains crucial in the formation of functional miRISC for miR-451. Deregulation of miR-451 expression occurs in many pathological conditions, especially neurodegenerations and in the course of carcinogenesis [52–54].

Some noncanonical miRNAs are derived from tRNA molecules, subjected to enzymatic cutting by hydrolases. DICER, ribonuclease 5, angiogenin (ANG), and other ribonucleases act on the cloverleaf structure of tRNA molecules. This results in the formation of tRNA-derived fragments (tDFs) and stress-induced fragments of 5′-tRNA and 3′-tRNA (tiRNAs). TDFs and tiRNAs, due to numerous analogies in structure and interaction on the genome, are identified as noncanonical mi-RNAs. Some subtypes of tDFs, formed into hairpin structures, can bind to AGO proteins on a similar basis to miRNA. In this form, they perform similar effector functions in the host genome. The preferential binding of tDFs to AGO proteins is likely to exhibit tissue specificity. In the human kidney embryonic cell line HEK-293, tDFs could bind with various proteins of the AGO family in variable proportions, dependent on the tDF subtype. This allows tDFs to perform the functions of miRNAs. tiRNAs (mainly 5′-tiRNAs) may reduce the translational activity of host cells. So far, there are no reports on the possibility of including tDFs and tiRNAs in complete RISC complexes. However, they are detected in P-bodies [40, 55–57].

Other ncRNAs that can be a source of noncanonical miRNAs are snoRNAs and, longer than them, scaRNAs, which are often described as a separate class of ncRNA. According to Jorjani et al., more than 80% of known human snoRNAs, found in various ribonucleoprotein complexes, recognize specific modification sites in RNA and participate in processes such as 2-O'-methylation or pseudouridylation of pre-rRNA, rRNA, and snRNA molecules [58]. Functions of other sno-RNAs, devoid of sequence specificity, allowing them to direct the processing of RNA (the so-called orphan snoRNAs), have not been fully understood. Most modern reports mention the possibility of binding orphan sno-RNAs or sca-RNAs by DICER or DGCR8 protein complexes. This results in the formation of so-called miRNAs that are derivatives of snoRNA or scaRNA (snoRNA/scaRNA-derived miRNAs, sno/sca-miR). Sno-miR and sca-miR can be recognized by the AGO1-AGO4 proteins and regulate genome expression in the same way as canonical route miRNAs [59–65].

## 3. Parkinson's Disease: An Overview and Pathogenesis

Belonging to the group of *α*-synucleinopathies, Parkinson's disease (PD) is the second most common neurodegenerative disease in the world (8-18 cases per 100000 persons annually). It is estimated that 0.3% of the Earth's population suffers from it. Within the age range of 60-80 years, PD may occur even in 1-4% of the patients. The median of the diagnosed cases is 60 years old, and the predicted lifespan of the patients is 15 years. Likelihood of the disease's occurrence is 1, 5-2 times bigger in men than in women. An intensification and rate of progression of motor symptoms are also lower in women. PD manifests itself mainly with bradykinesia, resting tremors, rigidity, and postural instability. Other symptoms are cognitive disorders, psychosis, gastrointestinal issues, or sleep disorders [26, 66, 67].

The causes of PD remain undetermined. As in many other diseases, the risk of PD's development is most probably the result of combined genetic and environmental factors. The most important recognized pathologies of the central nervous system (CNS) in the course of PD are degenerative changes and gradual loss of the dopaminergic neurons (up to 70%), especially in the midbrain's *substantia nigra pars compacta*. In the microscale, in the cytoplasm of affected neurons, the Lewy bodies can be observed. They are the acidophilic, insoluble, fibrillated aggregates, created by low-molecular proteins. The most common substrates here are *α*-synuclein and ubiquitin. The Lewy bodies are not pathognomonic for the PD, though. They may also appear in other neurodegenerations, i.e., in dementia with Lewy bodies (DLB) [68, 69]. *α*-Synuclein can be accumulated in neurons in other forms (i.e., oligomers). The aggregates and the other stressors combined trigger an immunological reaction in the *substantia nigra pars compacta*. This results in dopaminergic neurons death in many ways, such as apoptosis, autophagy, necrosis, or necroptosis. Also, the dysfunction of intracellular mechanisms, such as proteasomal degradation, lysosomal or antioxidative systems, and mitochondrial pathways, may lead to the development of PD [70–74]. There is a direct link between some autoimmune diseases and increased risk of PD. Some viral factors, i.e., human papillomavirus (HPV), may stimulate and induce of autoimmunological reactions against the neurons in the *substantia nigra pars compacta*, probably in the *α*-synuclein molecular mimicry-dependent manner [75, 76]. Releasing of proinflammatory cytokines may lead to the activation of microglia and consequently, to the changes in the phenotype of residual macrophages and astrocytes. This results in a loss of neuroprotective properties of microglia [77, 78]. An intestinal dysbiosis is a common feature of PD patients. Some species of a human microflora, i.e., *P. mirabilis* may be an additional source of endogenous *α*-synuclein. Protein can be next translocated to the brain, which was confirmed in mice [79–81].

Different gene mutations have been identified in at least 5-15% of PD patients. They can be inherited both in an autosomal dominant and autosomal recessive manner. Genes, which are related to the autosomal dominant inheritance of PD are PARK1 (SNCA), PARK4, PARK5, PARK8 (LRRK2), PARK13, and UCHL1, whereas PARK2 (PRKN), PARK6 (PINK1), PARK7, PARK9 (ATP13A2), or DJ-1 are inherited autosomally recessively [82, 83].

CNS is known as the most abundant source of miRNAs in a living organism. They are engaged in the regulation of the expression of many genes, which are vital for keeping the intracellular homeostasis in many stages of a cell's life. Excessive or decreased expression of the individual miRNA transcripts is directly linked with many diseases, including neurodegenerative disorders. Many miRNAs' functions have been determined in the potential context of pathological processes in dopaminergic neurons due to PD. Changes in specific miRNA concentrations, which could be determined in the most noninvasive way possible and which would correlate with the risk of occurrence or with the level of progression of the disease, could be a useful diagnostic tool in the future. miRNAs could also potentially be used in a different replacement therapy. These possibilities are especially important in diseases, in which the diagnosis and treatment are still lacking, e.g., neurodegenerative disorders [84–86]. This section includes present literature reports, regarding the dysregulation of the miRNAs in PD.

### 3.1. Microglia and Parkinson's Disease

Glial cells make up for up to 66% of the mammal's brain mass. 15% of these are microglia cells. In a physiological condition, microglia remain in a resting state. Macrophages of the microglia are involved in the elimination of infectious factors, dead CNS cells, and during a neuronal tissue remodeling. Microglia activation by the stressors, mainly in the Toll-like receptor- (TLRs-) dependent manner, leads to the development of different macrophage phenotypes, namely, M1 and M2. M1 is known for its proinflammatory properties, whereas M2 has been characterized as an immunosuppressive and neuroprotective phenotype. Proinflammatory cytokines and chemoattractants, released by the M1 in response to different stimuli, together with accompanying impairment of the M2 anti-inflammatory functions both can aggravate inflammatory reactions in CNS. As a result, gradual degeneration of a brain tissue and its functional impairment occurs, as it can be observed in PD [87–92].

Excessive activation of microglia during PD has been proven both in animal models and clinical trials. The expression of major histocompatibility complex II (MHC II) particles of the macrophages is increased. Knockout of the MHC II gene in the mice model of PD decreases leukocyte migration, reduces inflammatory process, and promotes the development of the M2 neuroprotective phenotype in microglia. Aggregates of the *α*-synuclein can be internalized and accumulated by glial cells through TLRs, mainly type 2. This facilitates the activation of the nuclear factor kappa-light-chain-enhancer of activated B cells (NF-*κ*B) factor pathway. Consistent stimulation of microglia leads to an increased amount of proinflammatory cytokines and chemotactic factors, such as TNF-*α*, IL-1*β*, or IFN-*β* being released. The NF-*κ*B stimulates inducible nitric oxide synthase (iNOS), which results in an intensified phagocytosis and microglial macrophages migration. Consequently, bigger amounts of *α*-synuclein are translocated further into the *substantia nigra pars compacta*. It promotes nigrostriatal neurons' death and PD progression. Due to a prolonging inflammation, fibroblast growth factor 20 (FGF20), mammalian target of rapamycin (mTOR), and early growth response factor 1 (EGFR-1) are being released. Through TLR type 4 receptors, these factors are able to additional NF-*κ*B activation, which further promotes death of the dopamine-producing neurons. Developing inflammatory state within the microglia and a subsequently disturbed cytokine signaling facilitates peripheral monocytes' migration to the CNS and their transformation into the macrophages. One of the signaling pathways, which can be altered in the course of PD is C-C motif chemokine receptor 2 (CCR2)/C-C motif chemokine ligand 2 (CCL2) signaling. There are, however, ongoing discussions, whether that abnormality promotes M1 or M2 transformation in PD patients [77, 93–101]. In one of the studies, carried out by Harms et al., *α*-synuclein-stimulated PD model was used. Switching off of the gene for a CCR2 receptor led in the experimental conditions to a decreased monocyte migration into the *substantia nigra pars compacta* and decreased microglia stimulation, which resulted in a neuroprotective effect [102].

Excessively released TNF-*α* may both directly (by inducing an inflammatory reaction) and indirectly promote a degeneration of dopaminergic neurons. That cytokine may stimulate enzymes, such as iNOS or cyclooxygenases, mainly COX-2. Subsequent increased release of a nitric oxide (NO) and prostaglandins further accelerates the inflammatory cascade. TNF-*α* is also able to create a synergistic effect with other proinflammatory factors, i.e., interleukin 1*β* (IL-1*β*). Basing on a positive feedback effect, the cooperation of these cytokines may lead to increased blood-brain barrier permeability and increased synthesis of adhesins and other inflammatory mediators. This facilitates further induction of the intracellular stress and neuronal cell apoptosis [103, 104].

Microglia environment contains inflammasomes, such as those associated with the nucleotide-binding oligomerization domain-like receptor protein 3 (NLRP3). Chronic effects of a stress factor trigger an activation of these protein complexes. This contributes to an increased expression of the proinflammatory IL-1*β* and IL-18 in glial cells. Excessive activity of the inflammasomes, activated by NLRP3, is characteristic in the *substantia nigra pars compacta* of PD patients. *α*-Synuclein aggregates, after being phagocyted by the microglial macrophages, may be a factor that releases an activation cascade in the NLRP3-associated inflammasomes. This results in a neuronal death. In the apoptotic processes, triggered by *α*-synuclein-mediated NLRP3 inflammasome activation, two proteins seem to play a crucial role, namely, the apoptosis-associated speck-like protein containing a CARD (ASC) and caspase-1 [105–108]. Switching off the gene for NLRP3, conducted in a mice model of PD treated with neurotoxin 1-methyl-4-phenyl-1,2,3,6-tetrahydropyridin (MPTP), resulted in a slower PD development in comparison to a control group [109]. In similar studies in mice, it was proven that the NLRP3 induction is an essential element of the *substantia nigra pars compacta* neurodegenerative process. There is also a link between the state of the activation of the NLRP3 inflammasome and the severity of motor symptoms, characteristic for PD. An improvement was especially noticeable after blocking of the IL-1 receptor [110]. A key role of the NLRP3 inflammasome in the context of PD neuropathogenesis seems to be interesting, especially while speaking of possible, future therapies of the disease.

### 3.2. miRNAs as a Microglia Activation Regulator and Neuroprotectors in PD

The mi-124 was characterized as a commonly occurring transcript in the CNS. Expression of the miR-124 substantially decreases in the MPTP-basing mice model of PD. The molecule has also shown an anti-inflammatory action during the PD progression. Repression of an inflammatory reaction in the microglia is linked with the miR-124's ability of silencing the genes, encoding proteins such as sequestosome1 (p62), and results in inhibition of p38 protein gene expression. These proteins are engaged in an autophagy and the NF-*κ*B activation, respectively. Administration of the exogenous miR-124 diminished an expression of p62 and p38. It also inhibited inflammatory processes in the midbrain's microglia of the MPTP-administered mice [111]. In a similar study, conducted by Yao et al., administration of the exogenous miR-124 led to a decreased activity of both the mitogen-activated protein kinase 3 (MEKK3), and the p65 transcription factor. It consequently resulted in a diminished NF-*κ*B pathway activation and slower microglial macrophages' activation [112]. The miR-124 deficiency correlated with an increased calpain 1 concentration. Calpain 1 can induce the autophagy and apoptotic processes via the p25/cdk5 signalization. The protein was characterized both in the context of a different tautopathies' patogenesis (such as AD or FTD) and the *substantia nigra pars compacta* degeneration in PD [113, 114]. Other proteins, which concentrations are increased with an accompanying decreased miR-124 expression, are the autophagy-promoting Beclin I and the microtubule-associated protein 1A/1B-light chain 3 II (LC3 II). Deficiency of the miR-124 transcript may also lead to an increased mammalian target of rapamycin and adenosine-monophosphate activated-protein kinase (AMPK and mTOR) signalization. Overactivity of these factors promotes a dopaminergic neuron loss via the autophagy and apoptosis [115]. The multifaceted role of the miR-124 in neuroprotection creates a possibility of using this transcript as a diagnostic or therapeutic tool in PD.

miR-155 is a molecule, which has been well-characterized in a literature and is known for its strong, proinflammatory activity [116, 117]. Thome et al. demonstrated that a deletion of the miR-155 gene in mice reduces an inflammatory response of the microglia against the *α*-synuclein inclusions and leads to a decreased MHC II and iNOS expression. Administration of a synthetic miR-155 resulted in the opposite effect; the inflammatory reaction against the *α*-synuclein was enhanced [118]. Decreased expression of the miR-155 results in an increased anti-inflammatory signalization, mediated by the Src homology 2 (SH2) domain-containing inositol polyphosphate 5-phosphatase 1 (SHIP1). As a consequence, both the recruitment of the NF-*κ*B factor and evolving of the macrophages towards the M1 phenotype are decreased. This may contribute to a neuroprotective effect [119]. TNF-*α*, which is released during an inflammatory reaction in the *substantia nigra pars compacta* may additionally induce an expression of the proinflammatory miRNAs. This applies to the miR-155, as well. It was suggested that an additive effect of the TNF-*α* and miR-155 may impair an expression of the genes for some mitochondrial complexes' subunits, namely, NADH: ubiquinone oxidoreductase core subunit V1. This contributes to an increased reactive oxygen species (ROS) concentrations and mitochondria damage, leading to an activation of apoptosis in the *substantia nigra pars compacta* neurons [120]. The other miRNA, known for its proinflammatory properties in the context of PD, is the miR-200a. It shows a complementarity with the 3′-UTR of a proapoptotic factor, Sirtuin 1 (SIRT1) mRNA. Cells, which were subjected to a stressor, the MPTP cation, were characterized by an overexpression of the miR-200a transcript and a decreased expression of the SIRT1. It further induced proapoptotic processes, which were linked with an activation of transcriptional factors, such as the forkhead box-containing FOXO and p53 [121].

As reported by Feng et al., an overexpression of the miR-330 may promote the SHIP1 regulation, consequently leading to a limited NF-*κ*B recrutation, decreased iNOS activity, and inhibited M1 phenotype polarization in the lipopolysaccharide- (LPS-) stimulated microglia [122]. Another miRNA, playing a role in the course of the PD-related inflammation is the miR-195. In an experimental model, with the use of the BV2 line, LPS-activated microglial cells, decreased expression of the transcript was observed. Transfection of the cells with the miR-195 caused an inhibition of the proinflammatory cytokine (namely, iNOS, IL-6, and TNF-*α*) production. Simultaneously, the expression of the anti-inflammatory factors, such as IL-4 and IL-10, was decreased. The Rho-associated kinase 1 (ROCK1), which is engaged in a proinflammatory signalization is regulated by the miR-195. Knockout of the ROCK1 gene elicited an anti-inflammatory effect in the BV2 cell line. The same effect was obtained after performing an induced miR-195 overexpression [123]. The interplay of the miR-195 and ROCK1 seems to play a significant role in the activation process of the microglial macrophages in the course of PD.

miR-let-7a is one of the pivotal regulators of signal transducer and activator of transcription-3 (STAT3) [122]. An intensified STAT3 signalization is inherently linked with the *substantia nigra pars compacta* microglia activation. This pathway seems to be involved in accelerating of the immunological response against the *α*-synuclein stressor. The expression of the miR-let-7a in PD mice in the experimental conditions was decreased. Induced overexpression of that molecule inhibited the microglia activation process, along with a decreased proinflammatory cytokines release in the BV2 cell line, in response to the *α*-synuclein aggregates. It was possibly accomplished through the STAT3 inhibition [124]. PD mice, after intracerebral miR-let-7a analogue administration showed an improvement of the motor functions [125]. It indicates a relevant function of that transcript in inhibiting of an inflammation and in preventing of the dopaminergic neuron death.

The miR-7116-5p may silence an expression of the TNF-*α*. Induced increase of the miR-7116-5p concentration in the microglia environment limits both its activation and the *substantia nigra pars compacta* neurons' atrophy [126]. miR-7 concentrations in PD patients seem to be constitutively impaired. Besides regulating of the SNCA *α*-synuclein gene expression, the miR-7 can reduce an activation of the NLRP3 inflammasome and the NF-*κ*B expression in PD-induced mice microglia [127, 128]. There is an ongoing discussion, whether the miR-7 could potentially be used in a replacement therapy in PD patients [129]. Similar characteristic is showed by the miR-190. Elevated levels of that transcript correlate with a limited proinflammatory cytokine (TGF-*β*, iNOS, IL-6) release. miR-190 is also able to downregulate the activity of NLRP3, as well as facilitating the dopaminergic neuron survival [130].

Increased expression of the miR-29c inhibits a proinflammatory factors release and inflammasome activation. It is achievable by silencing of the NF-kB and the other proinflammatory transcription factor, namely, the nuclear factor of activated T-cells 5 (NFAT5), as presented in the BV2 cell line [131]. Impaired expression of the miRNA-29 family molecules characterized a group of 80 PD patients and was strictly correlated with a level of disease progression [132]. It was proven that the microglia of toxin-induced PD mice show a substantial decrease in the miR-30e concentrations. The miR-30e injections were able to downgrade both the *α*-synuclein and proinflammatory mediator production. Transcript can also be directly involved in the NLRP3 mRNA silencing [133].

## 4. miRNAs and the PD's Genetics

An epigenetic regulation, in which the miRNAs are one of the most pivotal elements, plays a key role also in the PD pathogenesis. Changes in the expression of various miRNA transcripts, which are linked to the PD etiology, have been characterized in reports from recent years (Table 1). Dysregulation of specific miRNAs, along with point mutations, frameshift mutations, missense mutations, or deletions in the course of PD seems to be very important in its pathogenesis.

### 4.1. SNCA

The SNCA gene transcript's product is the *α*-synuclein, one of the most important proteins which have been yet characterized in the context of the PD-related *substantia nigra pars compacta* degeneration. In physiological conditions, the *α*-synuclein is engaged in a neuronal signalization regulation and synaptic vesicle recycling and participates in neurotransmitter synthesis, release, and storage. Aberrantly folded *β*-sheet structures, created by the *α*-synuclein, can emerge due to the SNCA mutations, environmental stressors, proteasomal dysfunction, or redox systems imbalance. Along with the other proteins, the unshapely *α*-synuclein is included in insoluble conglomerates in the *substantia nigra pars compacta* [134].

Among miRNAs, which may join the 3′-UTR of SNCA's mRNA and consequently regulate its expression, a few transcripts have been mentioned in a literature. These are, namely, miR-7, miR-153, or miR-203-3p. A deficiency of the miR-7 in the *substantia nigra pars compacta* is a hallmark in the course of PD. Increased miR-7 and miR-153 expression was correlated with decreased amounts of the SNCA mRNA transcripts in a cortical neuron culture. The *α*-synuclein concentrations are substantially increased after switching off the miR-7 activity. Currently, it is suggested that the miR-7 is involved in the SNCA's translation initiation inhibition, whereas the miR-153 induces a nuclease-mediated enzymatic degradation of the SNCA's mRNA [135–137]. Simultaneous transduction of the miR-7 and miR-153 into a cortical neurons culture, which had been previously treated with an active MPTP metabolite, resulted in an increased percentage of survived cells. It was possible due to an induction of the antiapoptotic BCL2-family protein, with a concomitant decrease in the caspase-3 concentration [138].

Shortage of the miR-203a-3p transcript was observed in the neuroblastoma SH-SY5Y cell line, treated with an MPTP. Administration of the toxin led to both a restricted proliferation of the cells and the promotion of their apoptosis. Increasing of the miR-203-3p concentration above normal levels made it possible to restore a normal SNCA gene expression. Another effect was an alleviated imbalance in pro and antiapoptotic factors in the SH-SY5Y cells. Other transcripts, which are able to regulate SNCA, include miR-30b, miR-34b/c, miR-214, miR-223, or miR-433 [139–142].

### 4.2. PINK1

According to reports, mutations in the PTEN-induced serine-threonine kinase 1 (PINK1) gene feature among patients with the early-onset (<50 years old), autosomal recessive PD [83, 143]. PINK1 proteins are mainly present in the outer mitochondrial membrane. Abnormalities in the PINK1 sequence result in an electron transport chain impairment and, therefore, in decreased ATP synthesis in a cell [144, 145]. The miR-27a and miR-27b are known as the PINK1 silencers. Proper activity of these transcripts prevents aberrant PINK1 proteins from being built into the mitochondrial membrane and its associated damage. This consequently inhibits a migration of the parkin protein into the mitochondria, therefore, counteracting their autophagy. The most important effect of that regulation is stopping the mitochondrial apoptosis cascade from being triggered, which results in a limited dopaminergic neuron death in PD [146]. Similar functions are attributed to the miR-140. Administration of the exogenous miR-140 led to decreased amounts of the PINK1 transcripts in neuronal cell cultures, as mentioned by Liang et al. [147].

### 4.3. PRKN

Similarly like in PINK1, mutations in the PRKN gene, product of which is parkin, are characteristic for the autosomal recessive, early-onset PD. A physiological role of the parkin (ubiquitin E3 ligase) is mediating in the autophagy, ubiquitination, and proteasomal degradation processes [144, 148, 149].

According to Zhou et al., the concentrations of parkin were decreased in mice after intracerebral MPTP administration. A comparable effect was obtained in the SH-SY5Y neuroblastoma cell culture, using an active cation of the toxin. Decreased amounts of parkin transcripts were accompanied by an increased miR-103a-3p expression. Switching off of the miR-103a-3p transcript activity, using an antisense nucleotide, induced an opposite effect. Thus, the miR-103a-3p may bind the 3′UTR of the parkin mRNA and modulate its expression. [150]. Both miR-181 and miR-218 are also able to regulate the PRKN gene expression. An increased amount of the miR-181a transcripts have an inhibitory effect on the parkin production. Knockout of the miR-181a-5p in rat C6 glioma cell cultures, treated with an MPTP cation led to an increase in antiapoptotic SIRT1 concentrations. Simultaneously, a decrease in lactic dehydrogenase (LDH) activity and a reduction of proinflammatory cytokine (IL-1*β* and TNF-*α*) concentrations were observed. The miR-181a transcript is also responsible for the SMAD1 and SMAD5 gene silencing. These genes are involved in a neuronal axon development. Knockout of the miR181a in vitro indirectly stimulated a growth in neuronal axial fibers. It was possible due to the increased levels of Smad 1 and Smad 5 proteins [151–153]. The miR-181-a-associated regulation seems to be a worthwhile therapeutic target.

High levels of miR-218 are correlated with a decreased parkin production, which consequently reduces a mitochondrial autophagy in the PINK-1-related manner. miR-218 may act neuroprotective by decreasing dopaminergic neuron damage in PD rats. That effect is achieved by inhibiting a cytoskeletal protein activity, namely, the cytoskeleton LIM and SH3 protein (LASP 1) by the miR-218 [154].

### 4.4. LRRK2

A product of the Leucine-rich repeat kinase 2 (LRRK2) gene is a protein, called dardarin. An activity of that kinase includes such processes as a cytoskeleton activation modulation, macrovesicle transport, or autophagy. There are functional connections known between dardarin and parkin. As a consequence of the LRRK2 mutation in the context of PD, many abnormalities may occur. This includes inducing of a dopaminergic neuron death, increased autophagy, or dendritic branching impairment [155–158].

Expression of the LRRK2 seems to depend on the miR-205 and miR-599. Decreased amounts of the miR-205 transcript have been described in the context of PD and were linked with an increased activity of the LRRK2 in neuronal cell cultures. Increased expression of the miR-205 in vitro leads to lower dardarin levels and vice versa [159]. A study on the miR-599 expression was conducted by Wu et al. An elevated activity of the LRRK2 in mice and cellular model of PD was accompanied by a decrease in the miR-599 concentrations. A correlation between the amounts of dardarin and the available pool of the miR-599 in a neuroblastoma cell line, treated with an MPTP cation, was similar as in the case of the miR-205 [160]. Botta-Orfila et al. observed a decreased expression of such miRNAs as miR-19a/b, miR-29a, and miR-29c in PD patients, with a coexisting point mutation G2019S in the LRRK2 gene, in comparison to healthy controls [161].

### 4.5. DJ-1

DJ-1, also known as PARK7, encodes a chaperone protein, which is responsible for reducing the *α*-synuclein aggregates formation. The DJ-1's activity seems to be inseparably linked to a redox balance of a cell [162]. An adequate reaction of chaperones to a presenting stressor reduces the accumulation of aberrantly structured proteins. DJ-1 mutations are rare (1-2% cases of the early-onset PD) and lead to an aggravated apoptotic process in the *substantia nigra pars compacta* [163].

DJ-1 is a target for the miR-494, which reduces its translation. Transfection of the miR-494 into the 3T3 fibroblasts culture resulted in a decreased DJ-1 protein concentration, along with increased concentrations of ROS. Induced overexpression of the miR-494 transcript in the MPTP-treated mice model was linked to a decreased DJ-1 activity. Changes at a molecular level were accompanied by an increased *substantia nigra pars compacta* neuron death. Motor dysfunction symptoms were also exacerbated [164, 165]. As reported by Chen et al., a group of 169 sporadic PD patients was characterized by increased miR-4639-5p concentrations in serum. This molecule is probably involved in the DJ-1 expression silencing. As in the case of the miR-494, the miR-4639-5p overexpression leads to an aggravated oxidative stress, as well as neuronal apoptosis in vitro [166]. Another exemplary miRNA, which is mentioned in the context of DJ-1 and PD, is miR-145-3p or miR-874. In one of the studies, their concentrations were elevated in PD patient's saliva [167].

## 5. Extracellular miRNAs in PD's Diagnostics

Different types of biological fluids contain ECmiRNAs, which are released by many types of tissues. Such miRNAs are most commonly bound with extracellular vesicles or protein complexes, i.e., with AGO proteins. Deregulation of different EC transcripts has been characterized in many diseases [168]. Given the fact that ECmiRNAs are highly stable in biological samples, they could become useful biomarkers in the future.

ECmiRNAs' profiles have also been studied in PD patients. The most commonly analyzed biological materials in these studies are serum, plasma, peripheral blood mononuclear cells (PBMCs), or saliva. These reports have defined an abundant pool of EC transcripts as possible PD biomarkers [132, 167, 169–181] (Table 2).

The exemplary transcripts are miR-144-5p, miR-200a-3p, or miR-542-3p, whose expression was increased in the CSF of PD patients [176]. Downregulated CSF miR-626 and upregulated plasma miR-105-5p could potentially be used in a differential diagnosis of PD and other neurodegenerations, such as Alzheimer's disease [169, 177]. Serum concentrations of some of the ECmiRNAs (i.e., miR-29a, miR-30c-5p, miR-132-3p, and miR-373) seem to correlate with a level of the PD's progression [132, 172].

From the perspective of PD's diagnostics, it is especially important to search for tools, which are able to detect the disease as early as possible. Currently, most of the PD cases are recognized in patients only after occurring of heavy motor symptoms, with accompanying substantial DA neuron loss [182]. In one of the studies, early-stage PD patients' CSF was characterized by decreased miR-27a-3p and miR-423-5p concentrations, together with increased Let-7f-5p concentration [178]. As showed by Dong et al., serum levels of miR-141, miR-146b-5p, and miR-193a-3p in early-stage PD patients were decreased in comparison with a control group [183]. Aberrant miR-27-3p family activity was detected in PD patients' PBMCs at an early stage of the disease [184]. In another study, 5 ECmiRNA transcripts showed a different activity exclusively in an early PD, compared to an advanced PD. Serum miR-29b-3p level was decreased, while 4 other transcripts' levels (miR-297, miR-346, miR-1909-5p, and miR-4462) were decreased [185].

Studies on ECmiRNAs in the context of PD diagnostics show some limitations, despite the results being promising. Most commonly, different studies compare different ECmiRNAs, often in different specimens. This consequently leads to a limited reproducibility of the results. Some other aspects, such as assay methodology, quality control, and suitable data processing also have to be improved when it comes to future ECmiRNA studies. Creating specific ECmiRNA panels, consisting of a few molecules at once, may improve a reproducibility, sensibility, and specificity of determinations.

## 6. miRNAs and PD's Therapeutic Perspectives

Regarding the fact that miRNAs are indispensable for a proper activity of the genome, they could be potentially considered as future therapeutics in many diseases. Overexpression of particular genes can be silenced using specific miRNA transcripts. Similarly, the anti-miRNA nucleotides (AMOs) are used to retrieve a proper activity of a downregulated gene. One of the most important issues here is to provide a suitable, safe carrier for these oligonucleotides. Two main issues are important here. The carrier should protect transcripts from being degraded by endogenous RNases. Additionally, in the context of PD, miRNA or anti-miRNA transporter should allow and facilitate the crossing of the blood-brain barrier (BBB). The most common carriers, used in studies on a therapeutic potential of miRNAs, are polymeric, lipid, and gold nanoparticles [186, 187]. Reports on the use of miRNAs as PD's therapeutics still remain scarce.

miR-124 is downregulated in both in vitro and in vivo PD models. Loss of dopaminergic neurons and neuroinflammation is reduced in the MPTP cation-treated SH-SY5Y cells or MPTP-treated mice [188]. As reported by Gan et al., miR-124 can be carried by polymeric nanoparticles, bound with rabies virus glycoprotein (RVG29). Such prepared transcript was stable in the circulatory system and was able to cross the BBB *in vitro.* In the same study, an intraventricular administration of miR-124-bound nanoparticles in MPTP-treated mice was performed. It resulted in MEKK3 inhibition, which consequently led to a decreased NF-*κ*B activation and reduced proinflammatory cytokines levels [189].

miR-124 is potentially able to stimulate a regeneration of the *substantia nigra pars compacta* neurons, which could be another interesting therapeutic approach in PD. Neurogenesis in adults is possible only in some regions of the CNS, i.e., the subventricular zone of the lateral ventricle [190]. As reported by Saraiva et al., in the subventricular zone primary cell culture, administration of miR-124-carrying nanoparticles stimulated a proliferation of the cells towards a neuroblast phenotype. Simultaneously, the amount of cells, proliferating towards an astrocytic phenotype, was decreased. It was achievable by inhibiting the expression of SOX9 and JAG1 genes by miR-124. In oxidopamine-treated mice, after an intraventricular injection of miR-124-bound nanoparticles, an increased proliferation of subventricular neuroblasts was observed [191].

## 7. Conclusion

The miRNAs are inseparable elements of an epigenetic activity regulation in the genome. Their activity depends on many proteins, which act as miRNA processors, transporters, and protectors. Irrespective of a pathway, in which both canonical and noncanonical transcripts come into being, they play a significant role in controlling of physiological processes and, once they have their production or function altered, could be associated to the emergence of pathologies. The vast majority of miRNAs occur in the CNS. miRNA transcript expression dysregulation has been described in many neurodegenerations, such as PD. Both extra and intracellular stressors may activate the microglia, which consequently leads to an impaired pro and anti-inflammatory signalization balance and triggers the degeneration of neurons in the *substantia nigra pars compacta*. In this review, we presented that these processes on many levels are accompanied by the miRNAs dysregulation, which has been proven in many both in vivo and in vitro studies. In the course of the PD, miRNA molecules may act both as neuroprotectors and pro-inflammatory factors, with a neuroprotective effect being rather more frequent in most studies. In pathological conditions, the products of some aberrant genes may aggregate and accumulate in the *substantia nigra pars compacta*. Some mutant proteins can also lose their chaperone activity or promote an apoptosis or autophagy in neurons, which contributes to the PD progression. Specific miRNA dysregulation is one of the hallmarks of the PD and has been widely described in many reports on PD patients. It has also allowed us to improve our knowledge regarding the PD pathogenesis. Experimentally induced overexpression or knockout of a particular miRNA may alleviate a dopaminergic neuron degeneration, which results in reduced motor symptoms of the PD. It seems reasonable to do more research regarding miRNAs engagement in the PD pathogenesis and their potential use in future therapies and diagnostic of the disease.

## Figures and Tables

**Table 1:** Exemplary genes, involved in PD's pathogenesis, and their specific miRNAs.

Gene	Product of the gene	3′-UTR mRNA's specific miRNAs
SNCA	*α*-Synuclein	miR-7, miR-153, miR-203a-5p, miR-30b, miR-34b/c, miR-214, miR-223, miR-433
PINK1	PTEN-induced serine-threonine kinase 1	miR-27a/b, miR-140
PRKN	Parkin	miR-103a-3p, miR-218, miR-181a-5p
LRRK2	Leucine-rich repeat kinase 2 (dardarin)	miR-205, miR-599, miR-19a/b, miR-29a/c
DJ-1	Protein deglycase DJ-1	miR-494, miR-4639-5p, miR-145-3p, miR-874

**Table 2:** ECmiRNAs' profiles in PD.

Analyzed material	Determination method	miRNA levels in PD patients		References
		Increased	Decreased	
Plasma	qRT-PCR	miR-105-5p	n/a	[169]
	qRT-PCR	miR-27a	let-7a/f and miR-142-3p	[170]
	qRT-PCR	miR-137	miR-222 and miR-124	[171]

Serum	qRT-PCR	n/a	miR-132-3p and miR-146-5p	[172]
	qRT-PCR	miR-30c-5p and miR-373	n/a	[173]
	qRT-PCR	n/a	miR-29	[132]
	qRT-PCR	miR-24 and miR-195	miR-19b	[174]
	qRT-PCR	miR-29c	n/a	[175]

CSF	qRT-PCR	miR-144-5p, miR-200a-3p, and miR-542-3p	n/a	[176]
	qRT-PCR	n/a	miR-626	[177]
	NGS	Let-7f-5p	miR-27a-3p and miR-423-5p	[178]

Saliva	qRT-PCR	miR-145-3p and miR-874	n/a	[167]
	qRT-PCR	n/a	miR-153 and miR-223	[179]

PBMC	qRT-PCR	miR-155-5p	miR-146a-5p	[180]

Plasma-derived extracellular vesicles	qRT-PCR	miR-30c-2-3p	miR-15b-5p, miR-106b-3p, miR-138-5p, and miR-338-3p	[181]

## Data Availability

No underlying data was collected or produced in this study.
